# 701 Impact of Alcohol and Methamphetamine Use on Burn Resuscitation

**DOI:** 10.1093/jbcr/irad045.177

**Published:** 2023-05-15

**Authors:** Connor Kenney, MD, Julie Rizzo, Elsa Coates, Maria Serio-Melvin, James Aden, Kevin Foster, Kareem AbdelFattah, Tam Pham, Jose Salinas

**Affiliations:** Brooke Army Medical Center, San Antonio, Texas; Brooke Army Medical Center, La Vernia, Texas; United States Army Institute of Surgical Research, San Antonio, Texas; United States Army Institute of Surgical Research, San Antonio, Texas; Brooke Army Medical Center, San Antonio, Texas; AZ Burn Center at Valleywise Health, Cave Creek, Arizona; UT Southwestern, Dallas, Texas; University of Washington, Seattle, Washington; United States Army Institute of Surgical Research, San Antonio, Texas; Brooke Army Medical Center, San Antonio, Texas; Brooke Army Medical Center, La Vernia, Texas; United States Army Institute of Surgical Research, San Antonio, Texas; United States Army Institute of Surgical Research, San Antonio, Texas; Brooke Army Medical Center, San Antonio, Texas; AZ Burn Center at Valleywise Health, Cave Creek, Arizona; UT Southwestern, Dallas, Texas; University of Washington, Seattle, Washington; United States Army Institute of Surgical Research, San Antonio, Texas; Brooke Army Medical Center, San Antonio, Texas; Brooke Army Medical Center, La Vernia, Texas; United States Army Institute of Surgical Research, San Antonio, Texas; United States Army Institute of Surgical Research, San Antonio, Texas; Brooke Army Medical Center, San Antonio, Texas; AZ Burn Center at Valleywise Health, Cave Creek, Arizona; UT Southwestern, Dallas, Texas; University of Washington, Seattle, Washington; United States Army Institute of Surgical Research, San Antonio, Texas; Brooke Army Medical Center, San Antonio, Texas; Brooke Army Medical Center, La Vernia, Texas; United States Army Institute of Surgical Research, San Antonio, Texas; United States Army Institute of Surgical Research, San Antonio, Texas; Brooke Army Medical Center, San Antonio, Texas; AZ Burn Center at Valleywise Health, Cave Creek, Arizona; UT Southwestern, Dallas, Texas; University of Washington, Seattle, Washington; United States Army Institute of Surgical Research, San Antonio, Texas; Brooke Army Medical Center, San Antonio, Texas; Brooke Army Medical Center, La Vernia, Texas; United States Army Institute of Surgical Research, San Antonio, Texas; United States Army Institute of Surgical Research, San Antonio, Texas; Brooke Army Medical Center, San Antonio, Texas; AZ Burn Center at Valleywise Health, Cave Creek, Arizona; UT Southwestern, Dallas, Texas; University of Washington, Seattle, Washington; United States Army Institute of Surgical Research, San Antonio, Texas; Brooke Army Medical Center, San Antonio, Texas; Brooke Army Medical Center, La Vernia, Texas; United States Army Institute of Surgical Research, San Antonio, Texas; United States Army Institute of Surgical Research, San Antonio, Texas; Brooke Army Medical Center, San Antonio, Texas; AZ Burn Center at Valleywise Health, Cave Creek, Arizona; UT Southwestern, Dallas, Texas; University of Washington, Seattle, Washington; United States Army Institute of Surgical Research, San Antonio, Texas

## Abstract

**Introduction:**

Mortality associated with burn injuries is declining with improved critical care, including resuscitation. However, patients admitted with concurrent substance use have increased risk of complications and poor outcomes. The impact of alcohol and methamphetamine use on acute burn resuscitation has been described in single center studies, however, has not been studied since implementation of computerized decision support for resuscitation. The purpose of this study was to evaluate resuscitation volumes for patients with alcohol and methamphetamine use within a large prospective observational trial at 5 major US burn centers.

**Methods:**

We performed an observational trial across five institutions with > 20% total body surface area (TBSA) burn, weighing >40kg that were resuscitated utilizing computerized decision support. Patients were evaluated based presence of alcohol, with a minimum blood alcohol level of 0.10, or positive methamphetamines on urine drug screen. Fluid volumes and urine output were examined over 48 hours and Wilcoxon Method was utilized to compare patient groups.

**Results:**

A total of 296 patients were analyzed. 37 (12.5%) were positive for methamphetamine use, 50 (16.9%) were positive for alcohol use, and 209 (70.1%) with negative for both. Patients positive for methamphetamine received a mean of 5.30 ±2.63 cc/kg/TBSA, patients positive for alcohol received a mean of 5.41 ± 2.49 cc/kg/TBSA, and patients with neither received a mean 4.33 ± 1.79 cc/kg/TBSA. Patients with methamphetamine or alcohol use had significantly higher fluid requirements than those who were negative for both substances. In the first 6 hours patients with alcohol use had significantly higher urinary output in comparison to patients with methamphetamine use which had similar output to patients negative for both substances.

**Conclusions:**

This study demonstrated that patients with alcohol and methamphetamine use had statistically significantly larger fluid resuscitation requirements compared to patients without. The effects of alcohol as a diuretic align with previous literature. However, patients with methamphetamine lack the increased urinary output as a cause for their increased fluid requirements. Methamphetamine’s neurologic and cardiovascular effects due to increased release of dopamine, serotonin, and norepinephrine are known. Further investigation is required to better understand the mechanism underlying the need for increased resuscitation after burn injury in patients positive for methamphetamines.

**Applicability of Research to Practice:**

The impact of alcohol and illicit substances on burn care, especially during the initial resuscitation, aids providers in guiding early critical care.

